# Assessment of Bisphenol A Levels in Preschool Children: Results of a Human Biomonitoring Study in Ankara, Turkey

**DOI:** 10.4274/jcrpe.galenos.2019.2019.0087

**Published:** 2020-03-19

**Authors:** İsmet Çok, Özlem Toprak İkidağ, Dilek Battal, Ayça Aktaş

**Affiliations:** 1 Gazi University Faculty of Pharmacy, Department of Toxicology, Ankara, Turkey; 2 Mersin University Faculty of Pharmacy, Department of Toxicology, Mersin, Turkey

**Keywords:** Bisphenol A, urine, children, liquid chromatography-mass spectrometry, Turkey

## Abstract

**Objective:**

There is general concern regarding environmental chemical exposure and the impact it may have on human health. This is particularly important for vulnerable populations such as infants and children during critical periods of development. Bisphenol A (BPA) is an endocrine disrupting chemical used worldwide over the last 30 years in many consumer products. Evidence points to widespread human exposure to BPA. The aim of this study was to evaluate the exposure of Turkish preschool children to BPA.

**Methods:**

This study was conducted as a preliminary investigation of BPA in urine, collected from 3-6 year old children living in Ankara. After spot urine samples were taken from preschool children, free BPA, β-D-glucuronide and total BPA were determined using high-performance liquid chromatography tandem mass spectrometry and adjusted by creatinine concentration.

**Results:**

Preschool children from Ankara (n=125; males n=70, females n=55; mean age: 4.50±1.26) were recruited. BPA was detected in 76.8% of children from Ankara city, with urinary concentrations ranging from < limit of quantification to 18.36 μg/g creatinine. Total BPA levels were not statistically different between boys (1.26 μg/g creatinine) and girls (2.24 μg/g creatinine) (p>0.05).

**Conclusion:**

This study is an important contribution to the limited information about childhood exposure to BPA. The estimated daily BPA intake in this study is substantially lower than the European Food Safety Authority derived tolerable daily intake of 4 μg/kg BW/day.

**What is already known on this topic?**

Bisphenol A (BPA) is an endocrine disrupting chemical and exposure to BPA is almost inevitable in daily life. Relationships between BPA exposure and various health risks have begun to be established. In different societies, BPA levels in young children seem to be higher in comparison with adolescents and adults.

**What this study adds?**

This first study of biomonitoring in preschool children from Turkey is an important contribution to the limited information about childhood exposure to BPA in Turkey and the world. The magnitude of exposure of BPA by children, estimated daily intake was calculated first time for Turkish children in this study.

## Introduction

There is general concern regarding environmental chemical exposure and its impact on human health, but this is particularly important for vulnerable populations, such as infants and children during sensitive periods of development. In 1997 the leaders of the G8 countries stated, “*We acknowledge that, throughout the world, children face significant threats to health from an array of environmental hazards. The protection of human health remains a fundamental objective of environmental policies to achieve sustainable development. We increasingly understand that the health and well-being of our families depends upon a clean and healthy environment. Nowhere is this more true than in the case of children, who are particularly vulnerable to pollution*” (1). In addition, one of the biggest concerns of the World Health Organization (WHO) for children is exposure to chemicals during the intrauterine and childhood periods and associated health problems that arise later in life (2). In recent years, exposure to environmental pollution with chemicals known to act as endocrine disruptors (EDs) has been implicated in the incidence of many diseases and disorders.

Bisphenol A (BPA), with approximately 3.6 million tons annual global production (3), is an ED. The United States Environmental Protection Agency (EPA) estimates that more than 400,000 kilograms of BPA are leached into the environment every year (4). Due to the widespread use of BPA, over 90% of tested humans have detectable BPA, with the highest levels found in infants and children (5).

Childhood exposure to BPA occurs through specific exposure routes including mouthing, food intake, the use of BPA-containing products, inhalation, dermal contact and ingestion. Children are more susceptible to chemicals such as BPA than the general population due to their rapid development and increased food intake per kg body weight (2). BPA exposure has been linked to a range of adverse human health outcomes including decreased fertility, behavioural effects, disruption of endocrine function, altered development and increased prevalence of metabolic diseases (4,5). For example, relationships between BPA exposure and altered neurobehavioral outcomes including hyperactivity, attention problems, anxiety, and depression, in children have been reported by several human studies (6,7,8,9,10,11). Following the demonstration of a wide variety of adverse effects associated with BPA exposure in humans and laboratory animals over the last two decades, the Canadian Ministry of Health banned the import and marketing of infant feeding bottles made of polycarbonate in 2008, as BPA is used in the production process. In 2011, the European Union banned BPA use in the production of polycarbonate baby bottles and prohibited the sale and import of BPA-containing products that come in contact with food for children aged 0-3 (12). The same restrictions have been applied in Turkey since 2011.

Numerous studies estimate exposures to BPA using urinary biomonitoring. Most have focused on adults from different societies to quantify human exposure to BPA. These studies have shown large variations between participants and studies, but very limited data are available for young children (13,14). To our knowledge, data regarding human exposure to BPA in Turkey are scarce (15). The primary aim of this study was to quantify exposure of preschool children to BPA.

## Methods

### Chemicals and Reagents

All chemicals used were of analytical grade. BPA and creatinine standards were purchased from Sigma-Aldrich (St Louis, MO, USA). The isotope-labeled internal standards ^13^C_12_-BPA (99%), creatinine-d3 and ^13^C_12_-BPA β–D-glucuronide (^13^C_12_-BPA-GLU) were obtained from Santa Cruz Biotechnology, Inc. (Santa Cruz, CA, USA). High-performance liquid chromatography (HPLC)-grade methanol and acetonitrile (KGaA, Darmstadt, Germany) were used. Ammonium acetate was obtained from J. T. Baker (Phillipsburg, NJ, USA). Stock solutions of the BPA (100 ng/mL) and BPA-GLU (1000 ng/mL) were prepared in methanol and were stored at -20 °C. Deionized water (18.2 MΩ) treated with the Millipore (Simplicity, 185) Milli-Q water purification system (Elga Labwater Veolia, Anthony, France) was used for all aqueous solutions.

### Study Population

Urine samples were collected from preschool children (3-6 years old) between November 2015 and May 2016. Four day-care centers in Ankara, Turkey participated. Each parent completed a questionnaire about their children’s dietary habits; exposure to BPA in their daily life, at home, and in the school environment; medical history; weight and height. The study was designed in accordance with the ethical standards of the Institutional and/or National Research Committee and with the 1964 Helsinki Declaration and its later amendments or comparable ethical standards. Approval for the study protocol was obtained from the Ethics Commission of Mersin University Clinical Research Ethical Committee in Mersin, Turkey (document number: 12.02.2015/37). Written informed consent was obtained from all parents of individual participants.

Each child provided a single spot urine sample collected in a 125 mL glass screw cap culture tube lined with polytetrafluoroethylene that had been previously cleaned with hexane. The sample was divided into aliquots. Urine samples from healthy children were collected in the evening, before a meal, to determine the spot urine concentrations of both free BPA and BPA-GLU.

The samples were kept cool until transportation to the study center and were immediately frozen at -20 °C until analysis.

### Sample Preparation

Sample preparation, chromatographic and mass spectrometric (MS) conditions were used as described previously (16). Briefly, ^13^C_12_-BPA was used as a stable internal standard and added to the samples at the beginning of the extraction. The BPA and BPA-GLU in 500 µL urine samples were purified by protein precipitation/dilution with 500 µL of acetonitrile and 50 µL of ^13^C_12_-BPA and ^13^C_12_-BPA-GLU. After protein precipitation, samples were centrifuged at 2250 rpm at 25 °C for 10 minutes. Total BPA values were calculated as reported previously. All other analytical data such as QA/QC assurance, matrix effects and data repeatability have been reported previously (16).

### Instrumental Analysis

Identification and quantification of free BPA and BPA-GLU were performed with an Agilent 1200 Series 6460 (Agilent Technologies, CA, USA) triple quadrupole MS with Jet-Stream atmospheric pressure electrospray ionization source and Mass Hunter data acquisition/Quantitation software. The HPLC system was equipped with a binary pump, vacuum degasser, low carryover autosampler and thermoregulated column compartment. Twenty microliters of the extract was injected onto an Agilent (Agilent Technologies, CA, USA) Pursuit 3 pentafluorophenyl propyl column (100×3.0 mm, 3 µm particle size). The mobile phases A and B consisted of 2 mM ammonium acetate in water and methanol respectively. The limits of detection for free BPA and BPA-GLU were 0.03 ng/mL and 0.10 ng/mL and the limits of quantification (LOQ) were 0.08 ng/mL and 0.33 ng/mL respectively. The tandem MS-MS was operated with negative electrospray ionization in the selected reaction monitoring (SRM) mode. Nitrogen was used as both curtain and collision gas. The monitored quantifier SRM transitions were 227.1>132.8 for free BPA, 403.1>113.1 for BPA-GLU, and 239.2>224.1 for ^13^C_12_-BPA (internal standard).

### Creatinine Analysis in Urine

Both the free BPA and BPA-GLU values obtained in this study were corrected for creatinine. To assess the impact of creatinine adjustment on the total variance of spot urine samples, urine creatinine levels were analyzed using a modified method developed and validated for creatinine analysis by Park et al (17). Briefly, a 10 µL aliquot of urine was diluted with milli-Q water (1000-fold) and 100 µL (5 mg/L) of creatinine-d3 (internal standard, 5 mg/mL) was added. Creatinine was analyzed with LC-MS/MS in electrospray positive ionization mode and the SRM transitions monitored were 114.1>86.1 for creatinine and 117.2>89.2 for creatinine-d3. One microliter of the extract was injected onto an Agilent (Agilent Technologies, CA, USA) Zorbax SB-C18 chromatographic column (3 x 50 mm, 3.5 µm particle sizes). The mobile phases A (water) and B (methanol) both contained 2 mM ammonium acetate. The analysis for creatinine was achieved using isocratic conditions (80%B).

### Estimated Daily Intake (EDI) Calculation

To understand the magnitude of BPA exposure in the children, EDI was calculated based on the assumption of urine excretion volumes of 0.4 L (ages 3-4 years) and 0.5 L (ages 5-6 years) for 24 hours for children (18). The daily exposure doses of BPA were estimated using the following equation:

$\overset{\mathrm{EDI}}{\left(\mathrm{\mu g}/\mathrm{kg}\;\mathrm{bw}/\mathrm{day}\right)}\;=\frac{\underset{\left(\mathrm{\mu g}/L)\;\;\;\;\;\;\;\;\;\;\;\;\;\;\;\;\;\;\;\;\;\;\;\;\;\;\;\;\;\;\;\;\;\;\;\;\;\;\;\;\;\;\;\;(L/\mathrm{day}\right)}{\mathrm{Urinary}\;\mathrm{BPA}\;\mathrm{concentration}\;x\;\mathrm{Urinary}\;\mathrm{output}}}{\mathrm{Body}\;\mathrm{weight}\;(\mathrm{kg})}$

### Statistical Analysis

The statistical evaluations of the data were performed with Statistical Package for the Social Sciences, version 11.5 for Windows (IBM Inc., Armonk, NY, USA). Data were summarized as minimum, maximum, median, mean, geometric mean (GM), and standard deviation for total and each group. The normality of the data distribution was assessed with the Shapiro-Wilk test. The Mann-Whitney U test was used for multiple comparisons between groups. A p value less than 0.05 were accepted as statistically significant.

## Results

**Figure 1:**
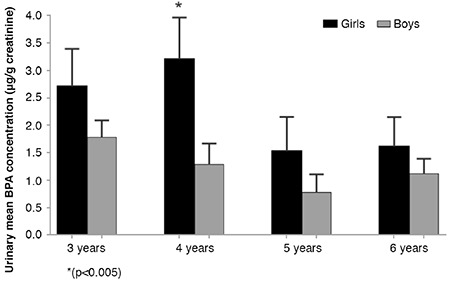
Urinary mean bisphenol A values obtained in age groups (μg/g creatinine)

In this study, free BPA and glucuronide conjugate of BPA (BPA-GLU) were measured in 125 preschool children (55 females, mean age 4.42±1.09 years and 70 males, mean age 4.56±1.39 years) who lived in Ankara. Table 1 presents the distribution of the main characteristics of the study populations. Urinary total BPA concentrations (adjusted for creatinine) in females and males are presented in Table 2. Total BPA was determined in 76.8% of the analyzed urine samples and BPA concentrations were equal to or above the LOQ of 0.08 ng/mL. Total urinary concentrations of BPA in Turkish preschool children ranged from LOQ-18.36 µg/g creatinine, with a mean concentration of 1.79 µg/g creatinine. The mean concentrations of total BPA in female and male groups were 2.24 µg/g creatinine and 1.26 µg/g creatinine, respectively, and there was no statistically significant difference (p=0.202). However, when the children were divided by age into <4 years and >4 years the mean BPA values of the <4 years-old females were statistically higher than the males of the same age (p=0.005) (Figure 1,Table 3).

For positive samples (values >LOQ) daily intakes ranged from 7 ng/kg bw/day to 2.916 ng/kg bw/day. The EDI for the preschool children was calculated as 35 ng/kg bw/day (GM) in this study. The mean EDI values were lower for the male group than the female group (Table 4), but this difference was not statistically significant (p>0.05). For risk assessment, in 1993, the US EPA (19) and in 2006 the European Food Safety Authority (EFSA) (20) recommended 50 µg/kg bw/day dose as the tolerable daily intake (TDI) and reference dose for BPA exposure. The EFSA revised the TDI for BPA to 4 µg/kg bw/day in January 2015 (21). The GM and 95^th^ percentile daily intakes of BPA determinated in both age groups and gender groups in this study were much lower than the guidelines established by the EFSA and US EPA (Table 4). This indicates that Turkish preschool childrens have a safe level of BPA exposure.

## Discussion

BPA is a high trade volume chemical because it is widely used in many consumer products and exposure is almost inevitable in daily life. In addition to being the first study to evaluate BPA exposure in preschool children in Turkey, the results of this study are important for providing basic data on BPA concentrations in the human population in Turkey. Studies assessing BPA exposure show that because of a dramatic increase in the use of BPA-containing products in daily life, BPA and its metabolites are present at detectable levels in nearly every person’s blood, tissue and urine. In order to assess the exposure of humans to BPA, measurement of their urinary concentration of free species, in this case BPA, and target compound conjugates, in this case conjugated BPA, is essential (22,23). BPA in biological samples is found as both free and conjugated BPA. Among the conjugated BPAs, BPA-GLU is a sufficiently specific and stable compound that can be regarded as a biomarker to evaluate BPA exposure (16). Varying levels of BPA and BPA-GLU are detected in urine samples depending on nutrition and lifestyle.

Although there is a general concern about possible effects of exposure to environmental chemicals on human health, these concerns are especially important for susceptible groups such as babies and children, during critical stages of their development. One of the biggest concerns of the WHO regarding infants is health problems that will show up later in life because of exposure to chemicals during the intrauterine and childhood periods. In particular, ED chemicals make important alterations in cellular pathways that provide a basis for these diseases (2). Hormones are the chemicals that regulate physiological homeostasis and functions of our body. These regimens are carried out in very small doses at the “picogram” level. Therefore, as a result of continuous exposure to EDs such as BPA, minor changes in hormone levels may cause major changes in biological function, particularly over the long term (2).

BPA is an ED (24) and is ubiquitous in the environment due to its widespread use in many consumer products globally over the past 30 years including in toys, baby bottles, plastic storage containers, heating containers for food and beverages, the lining of metal cans, medical equipment, consumer electronics and dental sealants, to give but a sample of the products containing BPA. A recent hypothesis states that BPA exposure may lead to many health risks (25), particularly obesity (26) and poor reproductive health (27). As exposure to this compound during a critical period, such as childhood, will provide a basis for exposure-related health problems, it is vital to determine the extent of BPA exposure in childhood both for the health of the individual and for future healthcare planning.

Numerous biomonitoring studies of children to quantifify childhood exposure to BPA during the last decade from different societies and different age groups have reported large variations between participants and studies. However, there are a limited number of studies of BPA exposure levels in preschool children. Huang et al (28) calculated the average global EDI of BPA for children based on the results of studies of children aged between 2-17 years from 18 nations between 2000 and 2016. The average global EDI for the children was 60.08 ng/kg bw/day in their study with the highest estimated child BPA daily intake found in Taiwan at 201.00 ng/kg bw/day, whereas Italy had the lowest with 15.34 ng/kg bw/day. The European Commission estimated the daily intake of BPA to be 0.4 µg/kg bw/day for adults, 1.2 µg/kg bw/day for children between 4 and 6 years, and 1.6 µg/kg bw/day for infants in EU countries (28). In our study, we detected half of the mean global value reported by Huang et al (28) (35 ng/kg bw/day).

Due to anticipation that exposure of children might be particularly high, recent studies have determined the BPA exposure extent in preschool children in various countries. Some examples of these studies are summarized in Table 5, with a focus on studies assessing preschool children, similar to our study. These studies demonstrate that BPA levels tend to decrease with increasing age in almost every society. For example, in a Health Measures Survey conducted in Canada between 2007 and 2011, the youngest study group, consisting of children aged 6-8, had the highest BPA level (Table 5) (7). Similarly, the 3-5 year age group (GM 3.55 µg/L) had a higher urinary BPA concentration than the 6-8 (GM 2.72 µg/L), 9-11 (GM 2.22 µg/L), and 12-14 (GM 2.42 µg/L) year age groups in the German Environmental Survey for Children (29). These results indicate that younger people, particularly infants and children below the age of six, are subjected to greater exposure risk. Similar results were obtained in our study.

In this study, no significant associations between the consumption of various canned foods and beverages and BPA levels were found (p>0.05) (Table 6). Urinary BPA levels of children consuming their food from heated plastic containers tended to be higher, but it was not statistically significant (p>0.05). Dental materials made of BPA derivatives such as BPA-dimethacrylate and BPA-diglycidyl-dimethacrylate, have been used as an alternative to mercury amalgams in dentistry. Therefore, in this study, whether the children had white dental filling was also evaluated. Composite restorations were not associated with urinary BPA concentrations in our study (p>0.05). Further, there were no statistical associations between BPA levels and the use of plastic materials and toys (p>0.05).

A few studies have determined the BPA exposure level of individuals in Turkey. In 2014, mean urinary BPA values were 0.61 µg/g creatinine in 200 people from Mersin city (15). In a further study, BPA amounts were quantified for 26 female children aged 4-8 years having the endocrine condition Idiopathic Central Precocious Puberty (ICPP) and 21 healthy controls. The average BPA concentration was 1.62 µg/g creatinine in the healthy group, whereas this value was 8.34 µg/g for the ICPP group (30). As a result, the estrogenic effects of BPA may be an etiologic factor for ICPP. Similarly, a study was performed on newly diagnosed ICPP patients (n=42; mean age 7.4±0.68 years) and peripheral precocious puberty (PPP) patients (n=42; mean age 7.4±0.61) between August 2012 and July 2013 in Ankara. Urinary median BPA levels were 10.60 µg/g creatinine for these ICPP patients and 10.15 µg/g creatinine for PPP patients and 10.91 µg/g creatinine for control group (31).

In a very recently completed study, BPA was detected in 100% of 40 maternal urine samples (GM; 0.12 µg/L), their 1–2-month-old infant urine samples (GM; 0.13 µg/L) and breast milk (GM; 0.12 µg/L). However, these BPA concentrations were relatively low compared to previous studies (32). In another recent study, urinary BPA levels of 50 children with type 1 diabetes mellitus and of 50 healthy children, all aged between 5 and 18 years, were measured using HPLC (38). In this study, urinary BPA levels of children with type 1 diabetes mellitus and healthy children were found to be 27.71±17.53 µg/g creatinine and 25.37±17.89 µg/g creatinine respectively. These values are somewhat higher than the values found in our study and other previous studies. This may be due to the HPLC method used to determine urinary BPA levels. Since HPLC is not a low-precision chromatographic method for determining BPA levels, it is not currently preferred by researchers to determine low BPA levels in biological materials.

### Study Limitations

We believe that the present study makes an important contribution to the limited information about exposure to BPA during childhood. Although 125 children from Ankara were included in this study, this number is not sufficient for this type of population biomonitoring study. However, our results might be evaluated as preliminary finding for Turkish children. In order to provide a better understanding of exposure to BPA, studies on a larger population are needed and daily exposure levels from different sources should be determined.

## Conclusions

From a global perspective, the average BPA exposure for children is much higher than in adult populations. Although regulations have attempted to prevent BPA exposure of food origin in infants and children, the extensive use of BPA in other commodities places humans in an environment with abundant levels of this chemical. Increasing knowledge of BPA-related toxicity in children indicates that exposure to BPA leads to health risks, especially in sensitive developmental periods. The determination of the exposure to BPA through biomonitoring, specifically in children, is very important for public health in all countries. This first study of biomonitoring in preschool children from Turkey should be followed by other studies from large communities from various provinces. Future studies should focus on children living in different provinces and extended follow-up of those exposed to high concentrations of BPA during critical developmental periods should be undertaken to evaluate the likely long-term impact of these EDs. It is believed that the results of this study will enhance awareness not only for BPA, but also for other chemical compounds, amongst health care professionals with an empahsis on pre-school aged children.

## Figures and Tables

**Table 1:**
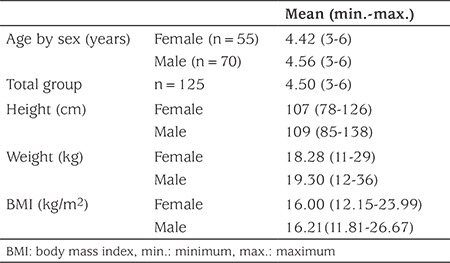
Population characteristics

**Table 2:**
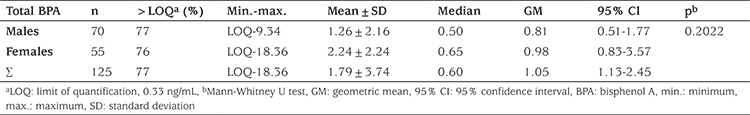
Urinary concentration of bisphenol A in Turkish preschool children (μg/g creatinine)

**Table 3:**
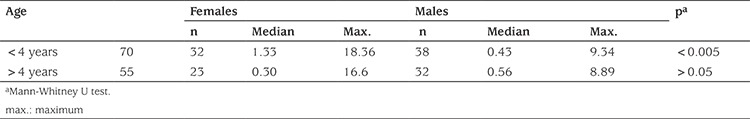
Urinary bisphenol A values by age groups in females and males (μg/g creatinine)

**Table 4:**
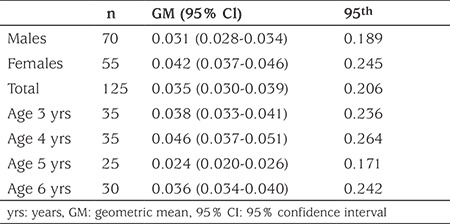
Estimated daily intake of bisphenol A in Turkish preschool children (μg/kg bw/day)

**Table 5:**
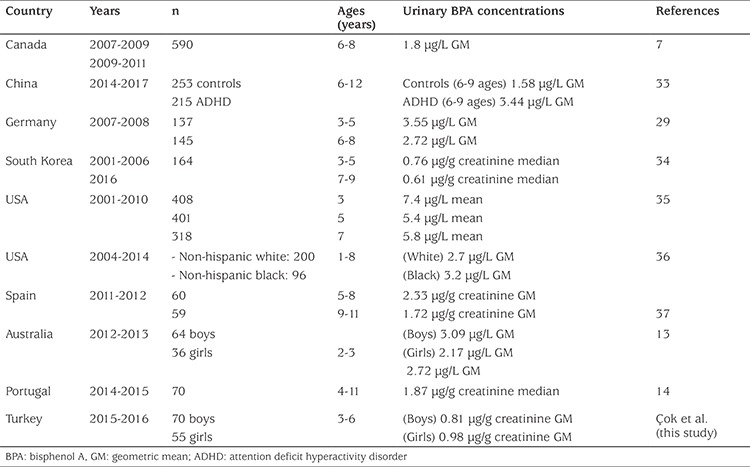
Urinary bisphenol A concentrations in children from different countries

**Table 6:**
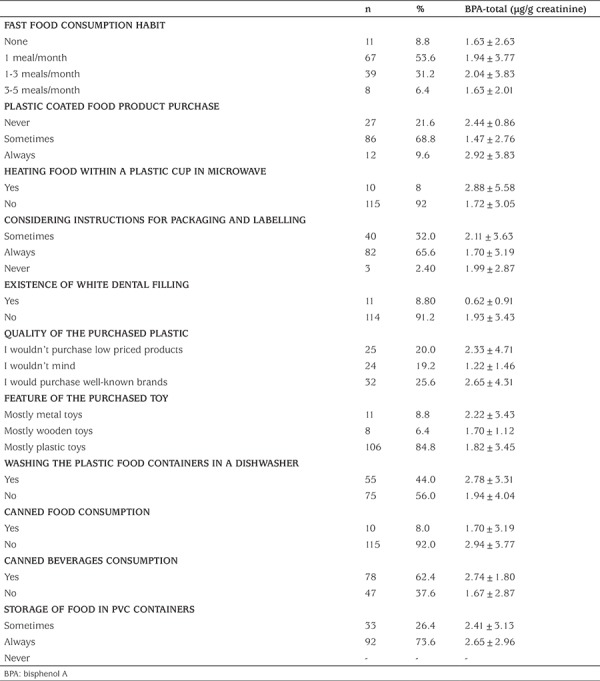
Bisphenol A-specific questionnaire data of the study population, mean±standard deviation
